# The Effect of Crystallinity on Compressive Properties of Al-PTFE

**DOI:** 10.3390/polym8100356

**Published:** 2016-10-11

**Authors:** Bin Feng, Xiang Fang, Huai-Xi Wang, Wen Dong, Yu-Chun Li

**Affiliations:** 1 College of Field Engineering, PLA University of Science and Technology, Nanjing 210007, China; fengbinplaust@gmail.com (B.F.); 15251853685@163.com (H.-X.W.); 2 The Equipment Research Institute of the Rocket Force, Beijing 10085, China; dwdongwen@126.com

**Keywords:** Al-PTFE, crystallinity, quasi-static compression, high strain rate mechanical properties

## Abstract

Al-PTFE (Al-polytetrafluoroethene) is an important kind of Reactive Material (RM), however only limited importance was placed to the effect of crystallinity of PTFE on the mechanical and reactive behavior. This paper investigated the influence of crystallinity on the compression behavior of Al-PTFE at strain rates range from 10^−2^ to 3 × 10^3^ s^−1^. Two kinds of samples were prepared by different sintering procedures to acquire different crystallinity. The samples’ crystallinity was characterized by the density method and X-ray diffraction method. The samples were tested using an electro-hydraulic press for quasi-static loading, and split Hopkinson pressure bars (SHPBs) for high strain rates. Low crystalline samples have consistently higher strength and toughness than the high crystalline samples. The phenomenon was explained by an “elastic-plastic network” model combined with the effect of chain entanglement density. A bilinear dependence of true stress on log $\dot{\varepsilon }$ was observed, and Johnson-Cook models were fitted separately according to the different strain rate sensitivity. Finally, a close connection between fracture and initiation of Al-PTFE was confirmed in quasi-static tests, SHPB tests, and drop weight tests. It was hypothesized that the high temperature at the crack tips of PTFE is an important promoting factor of initiation.

## 1. Introduction

The Al-PTFE (Al-polytetrafluoroethene) composite serves as an important benchmark for current development of Reactive Materials (RMs), or impact-initiated materials, which have many possible uses in military and civilian fields as structural reactives for target damaging or propellant/explosive additives [1,2]. Although the compressive properties of Al-PTFE across a range of temperatures and strain rates have been investigated in some literature [3,4], very little importance was placed on the processing history of the material prior to testing. However, the processing parameters (mixing method, molding pressure, sintering temperature, cooling rate, etc.) can greatly affect the crystallinity of PTFE, which is known to influence the deformation and fracture behaviors [5,6,7]. Moreover, Feng et al. [8] reported that low crystallinity Al-PTFE would initiate upon quasi-static compression, which may pose a great threat to the safety of manufacturing and handling of the material.

PTFE is produced by DuPont under the trade name Teflon and is known for its great stability and chemical inertness; however, upon decomposition PTFE releases volatile fluorinated gases that can produce highly exothermic reactions when in contact with metal particles [9]. PTFE is a complicated semi-crystalline material that undergoes the first-order crystalline transition at 19 °C from a well-ordered triclinic structure in phase II to a helical conformation in phase IV by the unraveling of molecular chains, and further rotational disordering and untwisting of the helices occurs above 30 °C giving way to phase I to form a pseudo-hexagonal structure, at ambient pressure [10]. PTFE is always treated like a composite that is composed of a “rigid” crystalline phase and a “softer” amorphous phase, where the amorphous regions are assumed to orientate along the maximum tensile stress while slip is seen to occur in the crystalline regions along parallel striations at low strains, and then overwhelmed by bowing or kinking about the striations at higher strains [11]. Recent real-time Raman spectroscopy [12] and X-ray diffraction [13] measurements shows that the amorphous phase dominates the elasto-viscoelastic regime of deformation, while the orientation of crystalline regions dominates the plastic flow and stress-hardening regime. It also shows that the fracture and failure was caused by lamellae destruction owing to molecular chain pulling-out mechanism, crystal tilting towards the stress direction, fragmentation, and void generation.

It is possible to obtain a wide variation in the degree of crystallinity by varying the heat history of PTFE. Until recently, only limited importance was placed to the effect of crystallinity on the mechanical properties of Al-PTFE [8]. However, several excellent research papers about the mechanical dependence on the crystallinity of pure PTFE may cast a light on the behavior of Al-PTFE, since PTFE often serves as the matrix in the composite to bear most of the exerted load. Koo and Andrews [14] conducted uniaxial quasi-static tensile tests on both a rapidlyquenched and slowcooled PTFE in the temperature range from −50 to 68 °C. They found the more highly crystalline PTFE is always more rigid than the less crystalline polymer at small deformations, but above 19 °C the less crystalline polymer shows a more rapid rate of “strain hardening”, which means at large strains lower crystallinity PTFE is stiffer. Rae and Brown [6] performed similar tensile experiments on PTFE with different crystallinity and the similar dependence on crystallinity is only observed at high-strains for elevated temperatures. To explain the PTFE’s behavior in tension, Koo and Brown both adopted the model which described the PTFE structure as an “elastic-plastic” network, in which crystalline domains are connected by elastic amorphous regions while the crystalline domains can flow plastically at sufficiently high stress or temperature. Rae and Dattelbaum [5] examined the behavior of PTFE with different crystallinity in quasi-static compression, at temperature of 0 °C, 24 °C, and 50 °C. The results show that more crystalline samples were consistently stronger, and a more surprising conclusion is that the rate of change with temperature was independent of the crystallinity. However, the maximum true strain in their experiments was only around 0.4, no data of high strain compression behavior of PTFE is available in the literature. Jordan et al. [15] compared the compression behavior of as-received PTFE and annealed PTFE, at strain rates range from 10^−1^ to 10^4^. They obtained opposite results to the study of Rae and Dattelbaum, i.e., the strength of lower crystalline samples was always higher. Even though the result agrees with studies performed on other crystalline polymers [16], they hypothesized that the effect of anisotropy played a much greater role than the crystallinity. Therefore, the effect of crystallinity on compression properties of PTFE requires further clarification. The influence of crystallinity on other mechanical properties, such as flexural, creep, dynamic, and fracture behaviors, could be found in [7,14,17,18].

In this paper, we investigated the effect of crystallinity of PTFE in Al-PTFE composite on mechanical and reactive behaviors in compression, at strain rates range from 10^−2^ to 3 × 10^3^ s^−1^.

## 2. Materials and Experiments

### 2.1. Sample Preparation

**Figure 1:**
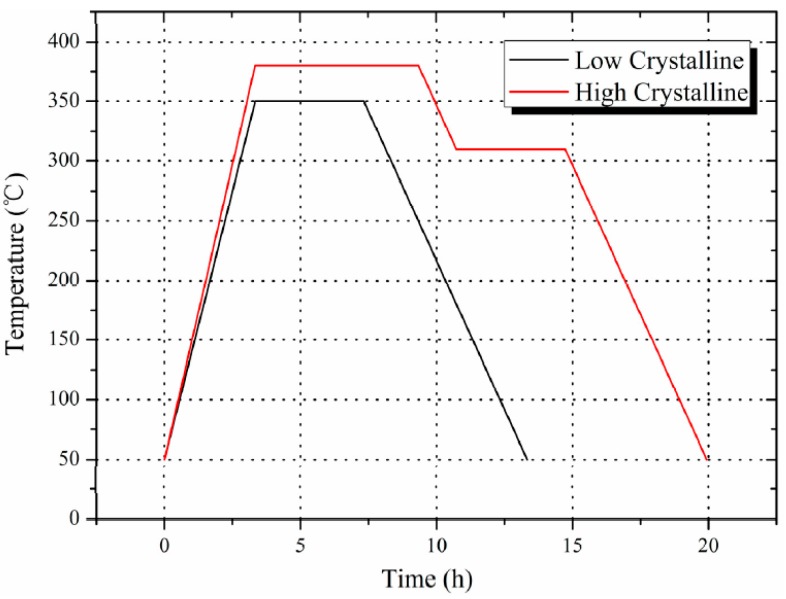
The sintering procedure of low and high crystalline Al-PTFE samples.

Cylindrical compression samples were prepared through a process including mixing, cold pressing, and sintering (based on the work of Nielson [19]). Firstly, Al (purity > 99.5%, 6–7 μm, from Jin Tian, Changsha, China) and PTFE (purity > 99.0%, 25 μm, from 3F, Shanghai, China) powders were suspended in an ethanol solution and mixed into a stoichiometric ratio (Al/PTFE, 26%/74%, by weight) by a motor-driven blender for 20 min, then dried at 50 °C for 48 h and cold pressed into cylinders under a compressive pressure of 60 MPa. Finally, pressed samples were put into a vacuum oven and different sintering procedures were adopted to obtain the varied crystallinity. As presented in Figure 1, the sintering procedure for low crystalline samples is as follows: heating to 350 °C in 2.5 h, holding for 4 h and cooling to room temperature at the rate of 50 °C/h. For high crystalline samples, the procedure is as follows: heating to 380 °C in 2.5 h, holding for 6 h, cooling to 310 °C at the rate of 50 °C/h and holding for 4 h, cooling to room temperature at the rate of 50 °C/h.

In the remainder of this paper, the lower-density samples sintered at 350 °C and the higher-density samples sintered at 380 °C will be designated as the “low crystalline” and “high crystalline” samples, respectively.

### 2.2. X-ray Diffraction Analysis (XRD)

In order to determine the degree of crystallinity of PTFE, Al-PTFE samples were characterized by a Rigaku D/max 2500/PC diffractometer (Rigaku, Tokyo, Japan) with Ni-filtered CuKα radiation generated at 40 kV and 20 mA. The diffraction intensity as a function of the angle 2-theta was measured between 0° and 60°. Prior to commencing the research, trial samples taken from the skin and the interior regions of one specimen were tested to detect any difference caused by temperature differences between the interior and exterior of the specimen during the sintering regime, and no statistical differences were discovered.Because of the high fracture toughness of Al-PTFE, the specimen should be immersed in liquid nitrogen before breaking it to expose the interior without experiencing significant plastic deformation. Therefore, XRD patterns of specimens at the skin region were chosen to calculate the crystallinity of PTFE for the sake of convenience.

### 2.3. Compression Experiments at Different Strain Rates

The specimens were tested across a range of strain rates from 10^−2^ to 3 × 10^3^ s^−1^, at room temperature. Given the ductile nature of PTFE, all strains referenced in this paper are true-strains. True-stress was calculated assuming a constant sample volume. The volume assumption was reported to be true at quasi-static strain rates by Rae and Dattelbaum [5] with data taken from two strain gages mounted on the sample, and it was also confirmed at high strain rate (~2300 s^−1^) by Jordan et al. [15] with laser diameter measurement and high-speed photographic recording in SHPB experiments.

Quasi-static compression was performed with a SFLS-30T electro-hydraulic press (MTS, Shanghai, China), and samples were nominally 10 mm diameter by 15 mm thick. Stress-strain curves were recorded automatically by the hydraulic press. In view of the naturally low coefficient of friction for PTFE against most materials, minimal sample barreling occurred even when deformed to 70% true-strain.

**Figure 2:**
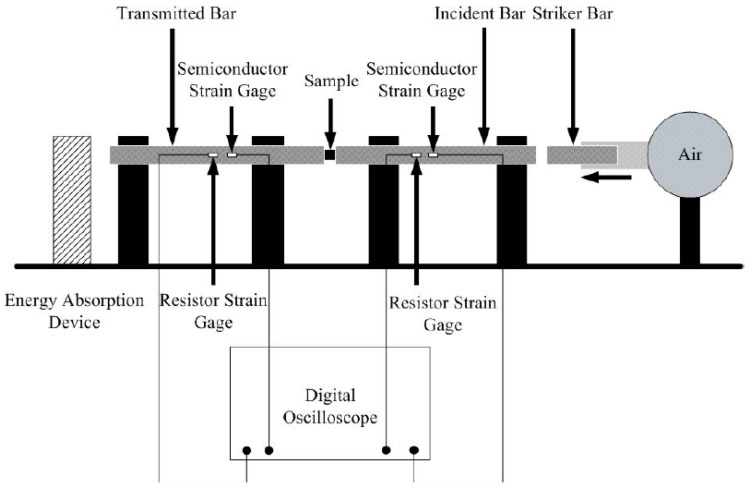
Schematic illustration of the SHPB system.

Compression experiments at high strain rates were conducted using a split Hopkinson pressure bar (SHPB), a schematic illustration of which could be seen in Figure 2. The SHPB system is comprised of a 6000 mm long, 20 mm diameter incident bar and a 3500 mm long transmitted bar with the same diameter, both of which are made of 6061-T6 aluminum or 440-HT stainless steel. The striker is 600 mm long and made of the same material as the other bars. In consideration of the low impedance of Al-PTFE, the lower-modulus aluminum bars was used to increase the signal-to-noise level. The samples were nominally 10 mm diameter by 5 mm thick, and the surfaces of which were lightly lubricated with petroleum jelly to reduce friction.

The properties of the sample are determined by measuring the incident, reflected, and transmitted strain signals, ε_I_, ε_R_, and ε_T_, respectively, using a set of semiconductor strain gages and a set of resistor strain gages. Semiconductor strain gages have a much higher gage factor (55 times than that of resistance strain gages), which were used to obtain data with higher signal-to-noise ratio. However, when the impact velocity of the striker bar is over 12 m/s, the input signal of semiconductor gages will over-range in the incident bar, and the signal of the resistor strain gages would be used instead. The two sets of strain gages were dynamically calibrated in situ by performing a number of impacts with measured striker bar velocities. From the measured impact velocity of striker bar v and the wave speed in the incident bar c_0_, the strain ε caused by the stress pulse can be determined and compared to the voltage output V from the strain gages to give a calibration in the form:

(1) $$\eta =\frac{\varepsilon }{V}=\frac{v}{2\times c_{0}}\times \frac{10^{6}}{V}$$

where η is the calibration factor.

Because of the low impedance of PTFE, the time required for a uniform uniaxial stress state to be achieved within the sample is much longer than metallic materials. Therefore, a pulse shaping technique was adopted. Carefully selected rubber shims with varied thickness was placed between the striker and the incident bar during impact, and the rise time of the incident wave pulse was increased to a value more comparable with the time to ring up the sample [20]. The effect of pulse shaping was analyzed in Section 3.2.

## 3. Results and Discussion

### 3.1. The Cystallinity of PTFE

The degree of crystallinity of the samples was quantified employing both density and XRD methods.

**Figure 3:**
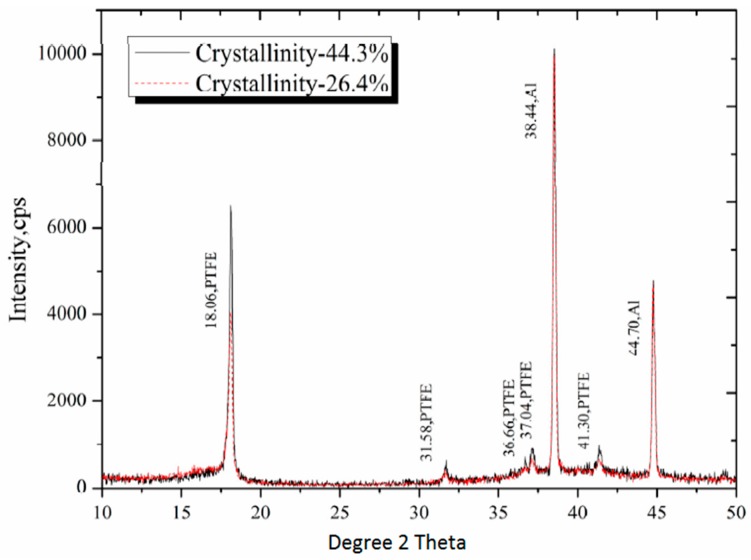
X-ray diffraction patterns of low and high crystalline Al-PTFE.

The X-ray diffraction results for crystallinity were obtained from diffraction patterns (Figure 3) by separating the reflections from the background by a fitting procedure [21]. Figure 3 shows that intensities of Al peaks in both specimens are identical, and the low crystallinity sample has lower intensity at all PTFE peaks. The degree of crystallinity was then determined by the equation:

(2) $$X_{C}=\frac{I_{C}}{I_{C}+KI_{A}}\times 100\%$$

where *I*_C_ and *I*_A_ are the scattered intensity for the crystalline and the amorphous phase, respectively. K is an adjustment factor, and the value was taken as 1.8 as reported by Sperati et al. [18]

The degree of crystallinity was determined from density measurements using:

(3) $$X_{C}=\frac{\rho_{c}}{\rho }\frac{\rho -\rho_{a}}{\rho_{c}-\rho_{a}}\times 100\%$$

where ρ is the measured density of the sample, ρ_c_ is the extrapolated crystalline density (2300 kg/m^3^) and ρ_a_ is the extrapolated amorphous density (2040 kg/m^3^). The constants are reported by Rae and Dattelbaum [5], who derived them as the averages of a wide range of reported values.

The calculated degree of crystallinity by both methods were listed in Table 1. The inconsistency between methods of calculating cystallinity in PTFE is close to that reported by Lehnert [22] and Hu [23], and all showed similar trends. In the density method, the values of ρ_a_ and ρ_c_ were extrapolated from experimental measurements, because PTFE cannot be manufactured as either purely amorphous or crystalline. And the density method is a much more empirical relationship and is sensitive to porosity and free volume as determined by the manufacturing process of PTFE. Therefore, the XRD method, which is relatively invariant on the manufacturing process, could be more accurate.

To verify that this difference in degree of crystallinity was achieved without any thermal degradation, samples from both sintering conditions were subjected to a second temperature cycle involving remelting and cooling at the same rate. The resultant crystallinity were essentially identical. This is an indication that there was no difference in molecular weight between the two samples [14].

### 3.2. Mechanical Characterization

**Figure 4:**
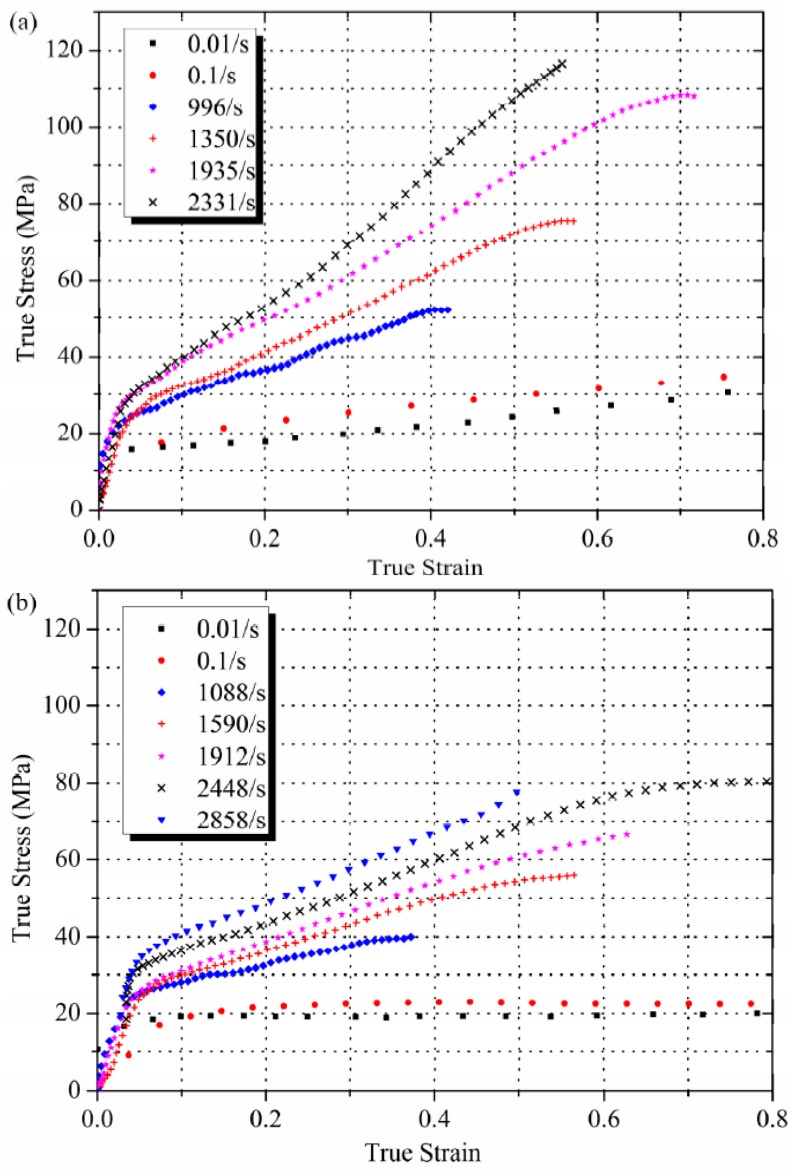
Stress-strain curves of (**a**) low crystalline and (**b**) high crystalline Al-PTFE tested in compression at room temperature.

Mechanical characterization experiments were performed on the low crystalline and high crystalline Al-PTFE samples at room temperature across a range of strain rates from 10^−2^ to 3 × 10^3^ s^−1^. The results are presented in Figure 4. Cai et al. [24] conducted dynamic compression experiments on pressed PTFE-Al-W mixture at a similar strain rate range, however, they used an unsintered specimen which has much lower strength and toughness than a sintered one. Moreover, the deformation mechanisms of sintered and unsintered PTFE are quite different because of the much less integrity of PTFE matrix in unsintered specimens.

**Figure 5:**
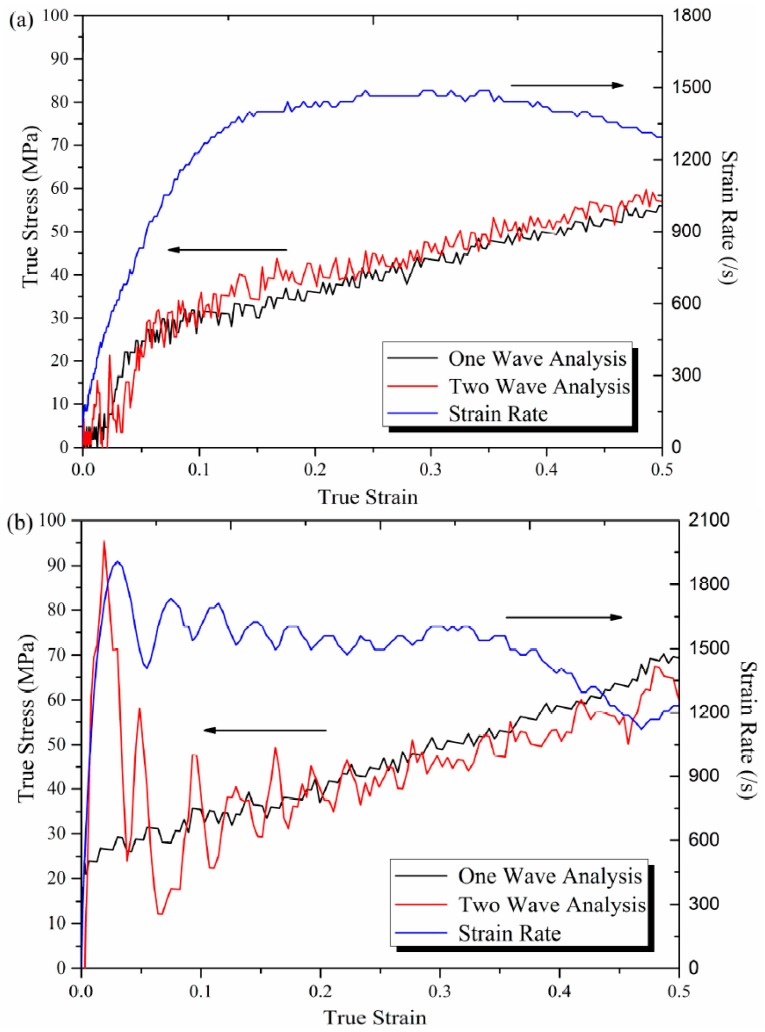
One-wave versus two-wave analysis and strain rate of Al-PTFE (**a**) with pulse shaping and (**b**) without pulse shaping.

The validity of SHPB tests was verified by examining the incident and transmitted pressure-bar data for stress-state equilibrium, as well as a constant strain rate. Stress-state equilibrium was verified by comparing the one-wave stress, determined from the transmitted pulse, and the two-wave stress, determined from the incident and reflected pulses, to determine if the two-wave stress oscillates around the one-wave stress [25,26]. The one-wave stress analysis reflects the conditions at the sample-transmitted bar interface and is often referred to as the sample back stress, and the two-wave analysis represents the conditions at the incident bar-sample interface (i.e., front stress) [18]. The stress-state equilibrium of high crystalline samples at the same strain rate of 1500 s^−1^, with and without pulse shaping, was compared in Figure 5. It can be seen that the front and back stress data exhibit very similar responses beyond ~0.05 strain for the test with pulse shaping, verifying that the sample attained a uniform stress state. However, for the test without pulse shaping, great oscillation was observed before 0.2 strain.

Comparing the strain rate curves, it can be seen that the strain rate reached constant more quickly in the test without pulse shaping, but the validity of data at early stage was greatly impaired by the non-uniform stress state, and severe attenuation of the strain rate was observed above 0.35 strain. For the test with pulse shaping, although the strain rate increased slower at the early stage, it reached constant soon after the sample attained the uniform stress state, and the attenuation is slight at high strain levels. Considering that the assumption of stress-state equilibrium should be satisfied in priority in SHPB tests, and the response of Al-PTFE at high strain levels is more important in the application of RMs, the test results with pulse shaping are more favourable.

**Figure 6:**
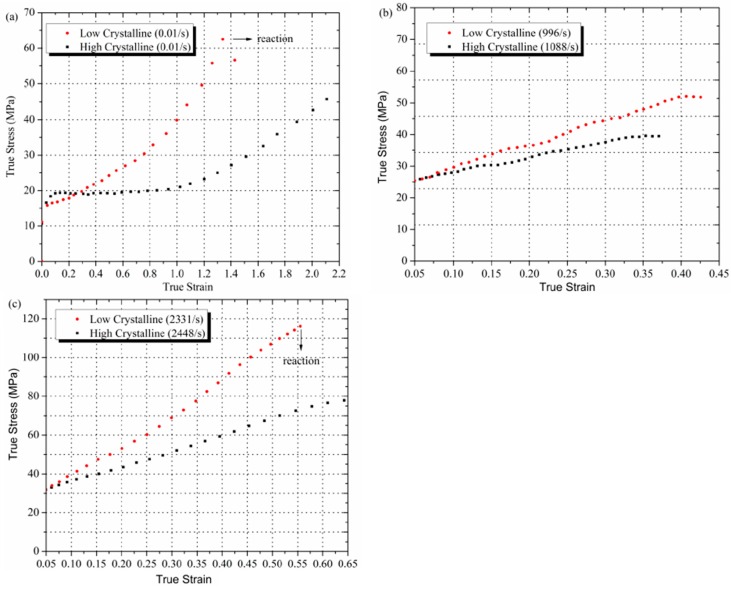
Comparison of stress-strain curves between low crystalline and high crystalline Al-PTFE at (**a**) strain rate of 0.01 s^−1^; (**b**) strain rate of about 1000 s^−1^; and (**c**) strain rate of about 2300 s^−1^. The point where the reaction took place is marked.

Comparison of the high crystalline and low crystalline Al-PTFE is presented for three strain rates in Figure 6. Please note that SHPB test results start from 0.05 strain, only from where stress-state equilibrium condition was satisfied. It can be seen that low crystalline samples have consistently higher strength than high crystalline samples, which agrees with studies on pure PTFE performed by Jordan et al. [15]. Rae and Dattelbaum [5] also examined the behavior of PTFE with different crystallinity in quasi-static compression at temperature of 0 °C, 24 °C, and 50 °C, at first sight they derived an opposite conclusion, the strength of low crystalline samples was slightly lower than that of high crystalline ones. However, the maximum tested strain in their work is only 0.45, but in our experiments a much higher true strain of 2.2 was reached. Actually, in Figure 5a the stress in the high crystalline sample is higher than the low crystalline one before 0.3 strain, which is consistent with Rae and Dattelbaum’s results. The difference between 0.3 and 0.4 strain could be caused by different moulding powder and heat treatment procedures adopted. Koo and Andrews [14] investigated the effect of crystallinity of PTFE by quasi-static tension tests at room temperature, and found that, at low strains, high crystalline samples were stiffer than low crystalline samples, while at higher strains, more amorphous samples were stiffer, which also supports the observation in Figure 5a. Koo explained this phenomenon with a morphological model referred to as an “elastic-plastic network”, in which the amorphous phase shows instantaneous rubbery elasticity, and the crystalline phase shows only plastic-yield deformation. When the low crystalline polymer is stressed, the crystalline regions can orient more freely and can more quickly reach a state of increased yield stress. In the more-crystalline polymer, this orientation is achieved more slowly because the crystalline regions, being larger and bulkier, are less free to move elastically. As the strain increases, little more deformation can be accommodated in the orientated crystalline regions and in low crystalline material the slip mechanism has been exhausted owing to the orientation. Therefore, at large strains lower crystallinity PTFE is stiffer than higher crystallinity material. However, this model neglected the effect of chain entanglement density, which was reported to be related to the strain hardening modulus in semi-crystalline polymers [27]. When PTFE crystalize from the melt, as in this case, rearrangement of molecular chains happens during chain folding. This “reeling in” of chains during crystallization leads to disentanglement of the chains from the melt [28], more pronounced for lower cooling rates, resulting in a lower entanglement density and, consequently, a lower strain hardening modulus in high crystalline samples. Combining the “elastic-plastic network” model with the effect of chain entanglement density, the experimental results in Figure 5 can be better explained. The “cross-over” of stress-strain curves was not observed in high strain rate results. It is hypothesized that there is not enough time for the different orientation speeds of PTFE affected by crystallinity at lower strain to fully manifest.

**Figure 7:**
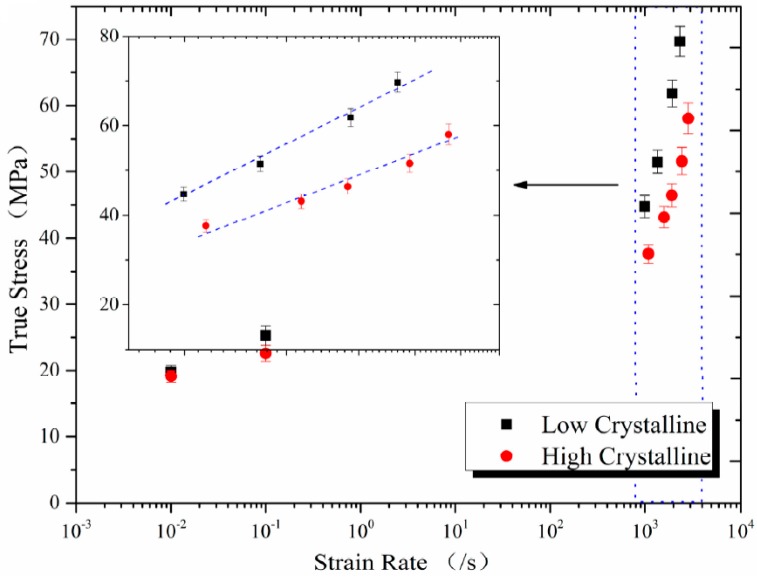
Comparison of true stress as a function of log (strain rate) between low crystalline and high crystalline Al-PTFE.

The strain rate effect of low and high crystalline Al-PTFE was compared in Figure 7 at a constant strain of 0.3. The data exhibit a bilinear dependence of true stress on log $\dot{\varepsilon }$, as reported by Jordan et al. [15] and by Walley and Field [29] on PTFE at the same range of strain rate. Walley and Field also observed some fluctuation between 10^−1^ and 10^3^ but they are not certain about the results because of the sparse data point in this range of strain rate. The bilinear dependence of true stress on log $\dot{\varepsilon }$ could be attributed to the time dependent microscopic processes accompanying deformation, such as the nucleation and growth of crazes, the orienting of molecular chains and crystalline grains, and the rotation and slip of crystalline regions. It takes time for these deformation mechanisms to fully manifest, but at the strain rate on the order of 10^2^ s^−1^ inertial and wave-propagation effects start to become important and these deformation mechanisms would be limited. It can be seen in Figure 7 that the strain rate sensitivity for low crystalline samples is consistently higher than high crystalline samples. On the other hand, the strain rate sensitivity was higher at high strain rates than at low strain rates for both low and high crystalline samples.

### 3.3. Application of the Johnson-Cook Model

**Figure 8:**
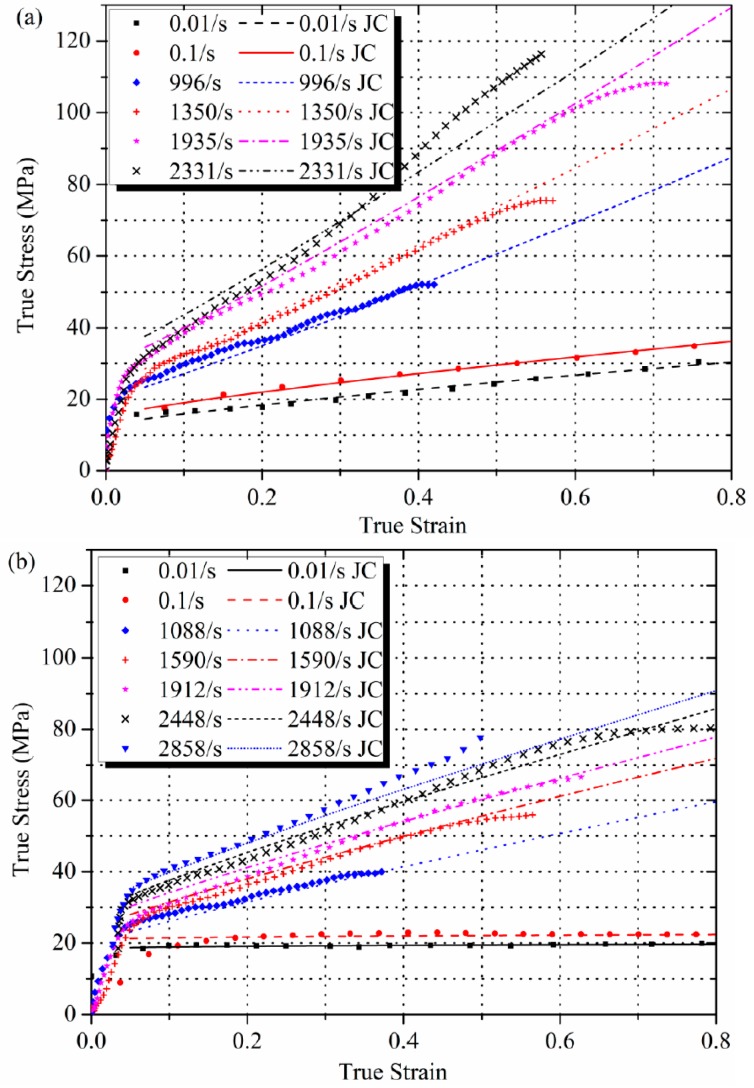
Comparison of Johnson-Cook model to (**a**) low crystalline and (**b**) high crystalline Al-PTFE experimental data.

The Johnson-Cook (JC) constitutive model was implemented and compared to the quasi-static and dynamic stress-strain curves in Figure 8. The JC equations are written as:

(4) $$\sigma =(A+B{\varepsilon_{p}}^{n})(1+C\ln(\frac{\dot{\varepsilon }}{{\dot{\varepsilon }}_{0}}))(1-T^{*m})$$

where σ, $\varepsilon_{p}$, $\dot{\varepsilon }$, and ${\dot{\varepsilon }}_{0}$ are stress, plastic strain, strain rate, and reference strain rate, respectively. A, B, C, m are material constants. Because all of the experiments were conducted at room temperature, the homologous temperature T* was set to 0. The fitting procedure was conducted separately for quasi-static and dynamic data, because the bilinear dependence of true stress on log $\dot{\varepsilon }$. Four sets of final parameters are given in Table 2.

Calculated JC curves agree well with the experimental data at low strain rates, but at strain rates over 2000 s^−1^ the model tends to underestimate the amount of strain hardening. Further experiments at higher strain rates need to be conducted to calibrate the model to fix the deviation.

Resnyansky et al. [30,31] proposed a constitutive model which takes the phase transition of crystalline PTFE into consideration; however the model was designed for pure PTFE and fundamental changes are needed to incorporate the strengthening effect of Al particles.

### 3.4. Reactions of Al-PTFE in Compression

Reactions of low crystalline Al-PTFE were observed in compression at quasi-static tests (0.01 and 0.1 s^−1^) and at dynamic tests (over 2300 s^−1^), the initiation point at stress-strain curves was marked in Figure 6a,c. However, no reaction was observed for high crystalline Al-PTFE at the tested range of strain rates. Nevertheless, it was confirmed by drop weight tests that the high crystalline Al-PTFE needs a much higher input energy (over 20%) to initiate. The details of the drop weight tests would be reported elsewhere.

**Figure 9:**
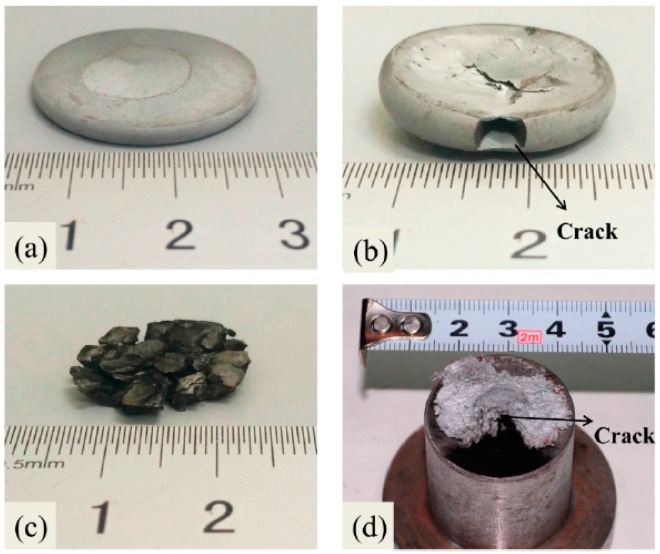
Recovered Al-PTFE samples after compression. (**a**) High crystalline sample after quasi-static compression; (**b**) Low crystalline sample after quasi-static compression; (**c**) Low crystalline sample after SHPB tests; (**d**) High crystalline sample after drop weight tests.

The reactivity of Al-PTFE was fundamentally related to its mechanical response [32,33]. One major difference in mechanical behavior between the low and high crystalline Al-PTFE is the markedly higher strength and toughness of low crystalline samples as presented in Figure 6. Another difference is that low crystalline samples are more prone to fracture in compression, as can be seen in Figure 9. In Figure 9a, a low crystalline Al-PTFE was compressed to a critical stress state, at which a crack developed at the periphery of the sample and flame came out from the crack and quenched, indicating the initiation site was in the crack. In Figure 9b, a high crystalline Al-PTFE was tested at the same force, but the sample deformed uniformly at all directions without any crack. The connection between initiation and cracking could also be confirmed by recovered samples in SHPB tests (low crystalline, Figure 9c) and in drop weight tests (high crystalline, Figure 9d), because black remnants (carbon black mostly), which are signs of reaction, could only be observed in cracks of recovered samples. The fact that low crystalline Al-PTFE was not initiated at strain rates lower than 2300 s^−1^ in SHPB test is because the low force and energy input of the incident bar at lower strain rates. It can be seen from Figure 4a that the stress-strain state at lower strain rates is lower than the initiation point, and little fracture was observed from the recovered samples.

Over many years, there has been much controversy about the mechanisms involved in the mechanical initiation of explosions. Although there is general agreement that initiation is due to “hot spots” where mechanical energy being converted into heat in localized regions by rapid or large deformation, the mechanisms of the formation of hot spots vary with different conditions of experiments. Walley et al. [34] noticed that because of the substantial fracture energies of polymers (typically ~100 Jm^−2^), sufficiently high temperatures may be achieved at the crack tips of polymers to initiate burning (deflagration) of neighbouring explosives. High temperatures at the crack tips of the polymer were confirmed by multiple instruments, including thermocouple techniques, temperature sensitive films, and infrared detectors [35,36,37,38]. Swallowe and Field [39] found that polymers could sensitize explosives upon impact, and the sensitizing effect was caused by hot-spots forming at crack tips and shear bands. Therefore, it is reasonable to consider the high temperature at the crack tips of PTFE as an important promoting factor of initiation. Furthermore, the rupture of covalent bonds during the cracking would release plenty of fluorine radicals which could readily react with Al particles at the temperature.

The yield and failure of semi-crystalline polymers could be triggered by multiple crazes or by localized shear yielding [40], and one of the mechanism often dominates the deformation process and dissipates energy. However, shear localization dissipates the energy more efficiently [41], which means less heat generated in the sheared region. It was also reported that the fracture toughness of PTFE exhibits a 42% decrease from pure mode-I (dominated by crazing) to near mode-II (dominated by localized shear) loading [42]. As discussed in [43], the crack induced reaction behavior of Al-PTFE under quasi-static compression was only observed in mode-I cracks (Figure 9b), which indicates that only the more energy-dissipating mode of deformation mechanism could cause the reaction.

## 4. Conclusions

This study investigated the influence of crystallinity on the compression behavior of Al-PTFE at strain rates range from 10^−2^ to 3 × 10^3^ s^−1^. Low crystalline samples have consistently higher strength and toughness than the high crystalline samples. The phenomenon was explained by a “elastic-plastic network” model combined with the effect of chain entanglement density. A bilinear dependence of true stress on log $\dot{\varepsilon }$ was observed, and Johnson-Cook model was fitted separately according to the different strain rate sensitivity. Finally, a close connection between fracture and initiation of Al-PTFE was confirmed in quasi-static tests, SHPB tests, and drop weight tests. It was hypothesized that the high temperature at the crack tips of PTFE is an important promoting factor of initiation.

## Figures and Tables

**Table 1:** Crystallinity of PTFE calculated by the density method and XRD method.

	Sintering temperature (°C)	Density (g/cm^3^)	*X*_c_ (Density method)	*X*_c_ (XRD method)
Low Crystalline Sample	350	2.25	43.2%	26.4%
High Crystalline Sample	380	2.30	63.7%	44.3%

**Table 2:** Johnson-Cook parameters derived from compression experiments for low crystalline and high crystalline Al-PTFE.

Parameter	Quasi-Static		Dynamic	
	Low crystalline	High crystalline	Low crystalline	High crystalline
A	20.41	18.00	24.51	23.80
B	22.19	1.79	104.82	58.52
C	0.07	0.06	0.59	0.45
n	1.35	0.30	1.09	0.88
${\dot{\varepsilon }}_{0}$	0.01	0.01	1350	1590

## References

[1] Wang H., Zheng Y., Yu Q., Liu Z., Yu W. (2011). Impact-induced initiation and energy release behavior of reactive materials. J. Appl. Phys..

[2] Glavier L., Taton G., Ducéré J.-M., Baijot V., Pinon S., Calais T., Estève A., Rouhani M.D., Rossi C. (2015). Nanoenergetics as pressure generator for nontoxic impact primers: Comparison of Al/Bi_2_O_3_, Al/CuO, Al/MoO_3_ nanothermites and Al/PTFE. Combust. Flame.

[3] Casem D.T. (2008). Mechanical Response of an Al-PTFE Composite to Uniaxial Compression over a Range of Strain Rates and Temperatures; DTIC Document.

[4] Raftenberg M., Mock W., Kirby G. (2008). Modeling the impact deformation of rods of a pressed PTFE/Al composite mixture. Int. J. Impact Eng..

[5] Rae P., Dattelbaum D. (2004). The properties of poly (tetrafluoroethylene) (PTFE) in compression. Polymer.

[6] Rae P., Brown E. (2005). The properties of poly (tetrafluoroethylene) (PTFE) in tension. Polymer.

[7] Brown E.N., Rae P.J., Bruce Orler E., Gray G.T., Dattelbaum D.M. (2006). The effect of crystallinity on the fracture of polytetrafluoroethylene (PTFE). Mater. Sci. Eng. C.

[8] Feng B., Fang X., Li Y.C., Wang H.X., Mao Y.M., Wu S.Z. (2015). An initiation phenomenon of Al-PTFE under quasi-static compression. Chem. Phys. Lett..

[9] Osborne D.T., Pantoya M.L. (2007). Effect of al particle size on the thermal degradation of Al/teflon mixtures. Combust. Sci. Technol..

[10] Brown E.N., Dattelbaum D.M. (2005). The role of crystalline phase on fracture and microstructure evolution of polytetrafluoroethylene (PTFE). Polymer.

[11] Speerschneider C.J., Li C.H. (1963). A correlation of mechanical properties and microstructure of polytetrafluoroethylene at various temperatures. J. Appl. Phys..

[12] Martin J., Ponçot M., Hiver J.M., Bourson P., Dahoun A. (2013). Real-time Raman spectroscopy measurements to study the uniaxial tension of isotactic polypropylene: A global overview of microstructural deformation mechanisms. J. Raman Spectrosc..

[13] Matsuba G., Ito C., Zhao Y., Inoue R., Nishida K., Kanaya T. (2013). In situ small-angle X-ray and neutron scattering measurements on a blend of deuterated and hydrogenated polyethylenes during uniaxial drawing. Polym. J..

[14] Koo G.P., Andrews R.D. (1969). Mechanical behavior of polytetrafluoroethylene around the room-temperature first-order transition. Polym. Eng. Sci..

[15] Jordan J.L., Siviour C.R., Foley J.R., Brown E.N. (2007). Compressive properties of extruded polytetrafluoroethylene. Polymer.

[16] Lee C.S., Caddell R.M., Yeh G.S.Y. (1972). Cold extrusion and cold drawing of polymeric rod: The influence on subsequent tensile and compressive mechanical properties. Mater. Sci. Eng..

[17] Sperati C., McPherson J. The effect of crystallinity and molecular weight on physical properties of polytetrafluoroethylene. Proceedings of the Meeting of the American Chemical Society.

[18] Sperati C.A., Starkweather H.W. (2006). Fluorine-containing polymers. II. Polytetrafluoroethylene. Adv. Polym. Sci..

[19] Nielson D.B., Tanner R.L., Lund G.K. (2003). High Strength Reactive Materials. U.S. Patent.

[20] Gray G., Kuhn H., Medlin D. (2000). Asm Handbook Volume 8: Mechanical Testing and Evaluation.

[21] Lee T.H., Boey F.Y.C., Khor K.A. (1995). X-ray diffraction analysis technique for determining the polymer crystallinity in a polyphenylene sulfide composite. Polym. Compos..

[22] Lehnert R.J., Hendra P.J., Everall N., Clayden N.J. (1997). Comparative quantitative study on the crystallinity of poly(tetrafluoroethylene) including raman, infra-red and ^19^F nuclear magnetic resonance spectroscopy. Polymer.

[23] Hu T.Y. (1982). Characterization of the crystallinity of polytetrafluoroethylene by X-ray and IR spectroscopy, differential scanning calorimetry, viscoelastic spectroscopy and the use of a density gradient tube. Wear.

[24] Cai J., Walley S., Hunt R., Proud W., Nesterenko V., Meyers M. (2008). High-strain, high-strain-rate flow and failure in PTFE/Al/W granular composites. Mater. Sci. Eng. A.

[25] Gray G.T.I., Blumenthal W.R., Trujillo C.P., Carpenter R.W.I. (1997). Influence of temperature and strain rate on the mechanical behavior of adiprene L-100. J. Phys. IV.

[26] Gray G.T. (2012). High-Strain-Rate Testing of Materials: The Split-Hopkinson Pressure Bar.

[27] Schrauwen B. (2003). Deformation and Failure of Semi-Crystalline Polymer Systems: Influence of Micro and Molecular Structure. Master’s Thesis.

[28] Peterlin A. (1980). Chain folding in lamellar crystals. Macromolecules.

[29] Walley S., Field J. (1994). Strain rate sensitivity of polymers in compression from low to high rates. DYMAT J..

[30] Resnyansky A.D., Bourne N.K., Millett J.C.F., Brown E.N. (2011). Constitutive modeling of shock response of polytetrafluoroethylene. J. Appl. Phys..

[31] Resnyansky A.D., Bourne N.K., Brown E.N., Millett J.C.F. (2014). Phase transition modeling of polytetrafluoroethylene during taylor impact. J. Appl. Phys..

[32] Zhang X., Shi A., Qiao L., Zhang J., Zhang Y., Guan Z. (2013). Experimental study on impact-initiated characters of multifunctional energetic structural materials. J. Appl. Phys..

[33] Sorensen B. (2015). High-velocity impact of encased AL/PTFE projectiles on structural aluminum armor. Procedia Eng..

[34] Walley S.M., Field J.E., Greenaway M.W. (2006). Crystal sensitivities of energetic materials. Mater. Sci. Technol..

[35] Fuller K.N.G., Field J.E. (1975). The temperature rise at the tip of fast-moving cracks in glassy polymers. Proc. R. Soc. A.

[36] Döll W. (1983). Optical interference measurements and fracture mechanics analysis of crack tip craze zones. Crazing in Polymers.

[37] Rittel D. (1998). Transient temperature measurement using embedded thermocouples. Exp. Mech..

[38] Zhang J., Jiang H., Jiang C., Cheng Q., Kang G. (2016). In-situ observation of temperature rise during scratch testing of poly (methylmethacrylate) and polycarbonate. Tribol. Int..

[39] Swallowe G.M., Field J.E. (1982). The ignition of a thin layer of explosive by impact; the effect of polymer particles. Proc. R. Soc. A Math. Phys. Eng. Sci..

[40] Meyers M.A., Nesterenko V.F., Lasalvia J.C., Xue Q. (2001). Shear localization in dynamic deformation of materials: Microstructural evolution and self-organization. Mater. Sci. Eng. A.

[41] Hoffmann T. (2003). Viscoelastic Properties of Polymers.

[42] Brown E.N., Rae P.J., Liu C. (2007). Mixed-mode-I/II fracture of polytetrafluoroethylene. Mater. Sci. Eng. A.

[43] Feng B., Li Y.-C., Wu S.-Z., Wang H.-X., Tao Z.-M., Fang X. (2016). A crack-induced initiation mechanism of AL-PTFE under quasi-static compression and the investigation of influencing factors. Mater. Des..

